# A thank you to all our reviewers

**DOI:** 10.1308/rcsann.2023.0112

**Published:** 2024-01-01

We would like to take this opportunity to thank you for the time and expertise that you generously contribute to reviewing articles submitted to the Annals and the Bulletin. We want to acknowledge your support during 2023, without which it would not be possible to preserve the high standards of our journals.

Abougamil, Ahmed
Achar, Priya
Acheson, Austin
Adcox, Max
Agu, Obiekezie
Ahmed, Ahmed
Al Saoudi, Tareq
Alanbuki, Ammar
Albani, Agustin
Alder, Louise
Ali, Stephen
Al-Lami, Ali
Allan, Anna
Allison, Callum
Almidani, Ammar
Ananth, Sankar
Anantharamakrishnan, Krishnan
Anderson, Iain
Aroori, Somaiah
Aruparayil, Noel
Ashton-Key, Margaret
Asif, Aqua
Attlmayr, Bernhard
Baker, Daryll
Baldwin, Alexander
Bance, Rujuta
Barnard, James
Barnes, Nicola
Basu, Narendra
Bater, Michael
Bath, Michael
Batten, Timothy
Battersby, Nick
Becker, Giles
Bedford, James
Beral, Dan
Berry, David
Bhardwaj, Rakesh
Bhutta, Mahmood
Biyani, C. Shekhar
Biyani, Shekhar
Blackburn, Julia
Body, Sam
Bolton, William
Booker, Simon
Boorman, Patricia
Bowley, Doug
Bowrey, David
Boyce, Tamsin
Branagan, Graham
Brearley, Stephen
Brewin, James
Britton, David
Brookes, Jocelyn
Brown, Chris
Brown, Peter
Brown, Steve
Buchanan, Gordon
Burke, Joshua
Busuttil, Andrew
Carey, John
Caruana, Edward
Cawrse, Nick
Cerovac, Sonja
Chambers, David
Chan, Anthony
Chan, David
Chan, Gareth
Chan, Katie
Chandler, Susan
Cheetham, Mark
Chinai, Natasha
Clifford, Rachael
Cook, Tim
Copson, Ellen
Cripps, Neil
Cubitt, Jonathan
Daniels, Ian
Danton, Mark
Davidson, Mike
Davies, Alun
Davies, Evan
Davies, Justin
Davies, Michael
Davies, Rhodri
Davis, Owen
Davis-Husband, Cameron
Day, Andrew
Dearing, J
Desai, Mital
Dhanda, Jagtar
Dhillon, Sukhrajbir Singh
Dick, Alastair
Dickson, Edward
Dilley, Christopher
Dimitriadis, Panos
Dimock, Richard
Dimou, Leonidas
Dobbs, Thomas
Donati-Bourne, Jack
Doran, Helen
Dubois, Richard
Duff, Sarah
Dunlop, Doug
Dyson, Peter
Eddama, Mohammad
El Boghdady, Michael
El-Gawad, Ahmed
Erete, Leanna
Evans, Jonathan
Fazal, Muhammad
Fearnhead, Nicola
Fischer, Jochen
Fleming, Myles
Fleming, Simon
Fligelstone, Louis
Frampton, Adam
Gambhir, Raghvinder
Ganau, Mario
Gandhi, Ashu
Gardiner, Matthew
Gaunt, Adam
Geh, Jenny
Gillespie, Patrick
Gilmour, Adam
Goddard, Jonathan Charles
Goldstraw, Miles
Grant, Stuart
Griffiths, Ben
Griffiths, Ewen
Gruber, Elizabeth
Guhan, Bala
Guy, Richard
Habib, Helai
Hamish, Maher
Hampton, Thomas
Hance, Julian
Hardie, John
Hardman, Gillian
Hargest, Rachel
Harries, Rhiannon
Harris, Guy
Harrison, Will
Hashmi, Faisal
Hau, Melinda
Hayter, Edward
Hendrickson, Susan
Heppell, Simon
Hill, Sue
Holcombe, Chris
Hone, Rob
Hu, Jennifer
Hugues, Adrian
Hussain, Amer
Hutton, Mike
Ibrahim, Lena
Inston, Nicholas
Irvine, Tracey
Jameson, Simon S.
Jiwani, Mateen
Jones, Alistair
Jones, Ceri
Jones, Oliver
Jones, Rhys
Joshi, Heman
Jowett, Billy
Kafai Golahmadi, Aida
Kannan, Ruben
Karaguannidis, Georgios
Kaushik, Robin
Kavia, Rajesh
Kenefick, Nick
Kenyon, Olivia
Kershaw, Steven
Khan, Mansoor
Khan, Tahir
Khong, Grace
Khorsandi, Maziar
King, Ian
Kinshuck, Andrew
Kirwan, Cliona
Knight, Hannah
Kokelaar, Rory
Kolocassides, Kyriacos
Koshy, Renol
Kothari, Prasad
Kudsk-Iversen, Soren
Kulkarni, Shrirang Vasant
Kumar, Nagappan
Lamb, J. N.
Lane, Ian
Lau, Andrew
Law, Tom
Lawes, Daniel
Leach, Paul
Leeder, Paul
Leinster, Samuel
Leslie, Ian
Lim, Chung
Loftus, Ian
Logghe, Heather
Loh, Christopher
Longman, Rob
Lovett, Bryony
Low, Willy
Luvhengo, Thifhelimbilu Emmanuel
Lyons, Marie
Machaal, Ali
Macklin, Chris
Magee, Conor
Mahendran, Hans
Malhas, Amar
Malik, Cat
Mallick, Sameer
Manfield, James
Mannion, Richard
Mannu, Gurdeep
Markham, Hannah
Massey, Lisa
Masters, Andrew
Maw, Andrew
Mazari, Fayyaz
McCarthy, Rebecca
McDermott, Frank
McIrvine, Andrew
McKinnon, Chris
Mehdian, Roshana
Miller, Peggy
Mirshekar-Syahkal, Bahar
Modi, Chet
Morgan, Jenna
Mount, Chloe
Mullan, Michelle
Mutimer, Jonathan
Nada, Hany
Nagala, Sidhartha
Nakas, Apostolos
Nash, Guy
Neal, Christopher P.
Nicol, Deborah
Nizam, Ikram
Nugent, Karen
Oddy, Mike
Odili, Joy
Oeppen, Rachel
Oliver, David
O'Leary, Fionnula
Paranathala, Menaka
Parsons, Simon
Patel, Anant
Patel, Kirtikbhai
Patel, Nimesh
Patel, Pranav
Patel, Rahul
Peckham-Cooper, Adam
Perrotta, Gerardo
Phillips, Alexander
Piper, Rory
Potter, Shelley
Poullis, Mike
Powell-Chandler, Anna
Price, Stephen
Pucher, Philip
Quinlan, John
Raghu, Ashley
Ramhamadany, Eamon
Rasheed, Ashraf
Rees, Rowland
Rehman, Syed
Rew, David
Richards, Toby
Robertson, Andrew
Robson, Andrew
Rockall, Tim
Ross, Alistair
Sagar, Peter M
Sarkar, Rupa
Sarma, Diwakar ryali
Saroglu, Azize
Saunders, Rick
See, Abbas
selvakumar, S
Shaaban, Abeer
Sharma, Anita
Sharpe, Ian
Shirley, Rebecca
Simpson, Gregory
Singh, Arvind
Skerritt, Clare
Slater, Michelle
Smith, C. D.
Smith, Frank
Smith, Katherine
Soliman, Faris
Stafford, G. H.
Stefanou, Demetrios
Stephens, Michael
Stephenson, Brian
Stevenson, Andrew
Stone, Chris
Sulaiman, Lutfi
Swarnkar, Keshav
Tatla, Taran
Taylor, Gregory
Tennyson, Charlene
Thornton, Michael
Torkington, Jared
Tozer, Phil
Travers, Hannah
Trickett, Ryan
Trompeter, Alex
Upile, Navdeep
Uppal, Harpal
van Laarhoven, Stijn
Vaughan, Casey
Vimalachandran, Dale
Viswanath, YKS
Vlastarakos, Petros
Voller, Tom
Wahba, Basim
Walijee, Hussein
Warren, Oliver
Warusavitarne, Janindra
Wassif, Hoda
Welchman, Sophie
Welsh, Fenella
Wheeler, James
Wijesinghe, Lasantha
Williams, Gethin
Williams, Nigel
Williamson, James
Williamson, Jeremy
Wood, Sandy
Yardley, Iain
Zaver, Vasudev
Zilidis, George

